# Systematic Review and Meta-Analysis of Carotid Artery Stenting Versus Endarterectomy for Carotid Stenosis: A Chronological and Worldwide Study

**DOI:** 10.1097/MD.0000000000001060

**Published:** 2015-07-02

**Authors:** Lei Zhang, Zhiqing Zhao, Yaoming Ouyang, Junmin Bao, Qingsheng Lu, Rui Feng, Jian Zhou, Zaiping Jing

**Affiliations:** From the Department of Vascular Surgery, Changhai Hospital, Second Military Medical University, Shanghai, China (LZ, ZZ, YO, JB, QL, RF, ZJ); and Department of Surgery, Changhai Hospital, Second Military Medical University, Shanghai, China (JZ).

## Abstract

There are disparities among the results of meta-analyses under different circumstances of carotid artery stenting (CAS) versus endarterectomy (CEA) for carotid stenosis. This study aimed to assess the efficacies of CAS and CEA for carotid stenosis at 5-year intervals and worldwide.

Comparative studies simultaneously reporting CAS and CEA for carotid stenosis with at least 10 patients in each group were identified by searching PubMed and Embase in accordance with preferred reporting items for systematic reviews and meta-analyses guidelines, and by reviewing the reference lists of retrieved articles.

The studies were stratified into different subgroups according to the publication year, location in which the study was mainly performed, and randomized and nonrandomized study designs.

Thirty-five comparative studies encompassing 27,525 patients were identified. The risk ratios (RRs) of stroke/death when CAS was compared with CEA within 30 d of treatment were 1.51 (95% CI 1.32–1.74, *P* < 0.001) for overall, 1.50 (95% CI 1.14–1.98, *P* = 0.004) from 2011 to 2015, 1.61 (95% CI 1.35–1.91, *P* < 0.001) from 2006 to 2010, 1.59 (95% CI 1.27–1.99, *P* < 0.001) in North America, 1.50 (95% CI 1.24–1.81, *P* < 0.001) in Europe, 1.63 (95% CI 1.31–2.02, *P* < 0.001) for randomized, and 1.44 (95% CI 1.20–1.73, *P* < 0.001) for nonrandomized comparative studies. CEA decreased the risks of transient ischemic attack at 30 d (RR: 2.07, 95% CI 1.50–2.85, *P* < 0.001) and restenosis at 1-year (RR: 1.97, 95% CI 1.28–3.05, *P* = 0.002). Data from follow-up showed that the RRs of stroke/death were 0.74 (95% CI 0.55–0.99, *P* = 0.04) at 1 year, 1.24 (95% CI 1.04–1.46, *P* = 0.01) at 4 year, and 2.27 (95% CI 1.39–3.71, *P* = 0.001) at 10 year. This systematic review, compared with those of other meta-analyses, included all available comparative studies and analyzed them at 5-year intervals, in different continents, and under different study designs. Current evidence suggests that the efficacy of CEA is superior to CAS for freedom from stroke/death within 30 d, especially from 2006 to 2015, in North America and Europe. Meanwhile, the superiority was also observed for restenosis at 1-year, transient ischemic attack within 30 d, and stroke/death at 4- and 10-year follow-ups.

## INTRODUCTION

Carotid stenosis is a major cause of ischemic stroke^1^ and it is estimated that ∼700,000 incidents are reported annually in the United States;^2^ therefore, the objective of carotid stenosis treatment is to reduce the risk of stroke or stroke-related death. Carotid endarterectomy (CEA) was introduced > 60 years ago as an effective approach to preventing stroke, and carotid artery stenting (CAS) has provided a less-invasive alternative in recent years;^3^ however, the results of previous meta-analyses that examined these protocols are ambiguous under different circumstances, and the therapeutic strategy of choosing between CEA and CAS is still a dilemma. Several studies have demonstrated that CAS is inferior to CEA because CAS increased the stroke or death rate within 30 d of treatment.^4–6^ Other studies have shown that CAS might be equivalent to CEA, especially in patients < 70 years old.^7–9^ In addition, the timeframes and regional discrepancies were not taken into account in previous meta-analyses.

In this meta-analysis, we systematically reviewed the current body of evidence comparing CAS with CEA in the treatment of carotid stenosis, and pooled the data for analyzing any stroke/death rate within 30 d at 5-year intervals, in different continents, and in randomized and nonrandomized comparative studies. We also pooled the data for analyzing restenosis, transient ischemic attack (TIA), and any stroke/death rates at different follow-up points.

## METHODS

The systematic review and meta-analysis was performed in accordance with the standards set forth by the statement from the Preferred Reporting Items for Systematic Reviews and Meta-Analyses.^10,11^ As this study is a systematic review and meta-analysis, ethical approval was not required.

### Data Sources and Search Results

The PubMed and Embase databases were searched from inception until February 4, 2015, restricted to studies in English and on humans. There were no restrictions on the year or the type of publication. The search strategy was amended for each database (see Table S1, Supplemental Content, which demonstrates the search strategies for PubMed and Embase databases). A hand search was also performed of all the references in the included studies for potential valuable and relevant publications.

### Study Selection

The inclusion criteria were as follows: (1) comparative study simultaneously reporting CAS and CEA for carotid stenosis; (2) at least 20 patients in the study and 10 patients in each group. Based on the guidelines, reviews, case reports, clinical trial protocols, commentaries/editorials, guidelines, new techniques/devices, restenosis therapy, basic and other research, systematic reviews and/or meta-analyses were excluded. The studies reporting only CEA or CAS were also excluded. After full-text articles were assessed for eligibility, the studies from Nationwide Inpatient Sample and New York and California States data were excluded. After qualitative syntheses, studies that were the same but were reported in different years were also excluded.

### Data Extraction

Two investigators (LZ and JZ) independently extracted data using a standard form. Disagreements were resolved by consensus. Data were extracted pertaining to any stroke/death, restenosis, TIA rates, and pooled for the main analysis according to the intention to treat principle.

### Outcome Measurement

The studies were stratified into different subgroups according to the publication year, the location in which the study was mainly performed, and the different study designs. The primary end points were any stoke/death rates within 30 d at 5-year intervals, in different continents, and in randomized and nonrandomized comparative studies. The secondary end points were restenosis rate at 1- and 2-year follow-up, TIA rate within 30 d and 1-year follow-up, and the stroke/death rate at 1-, 2-, 3-, 4- and 10-year follow-up points.

### Methodological Quality

The Cochrane Collaboration risk of bias tool was used to assess the quality of included randomized controlled trials (see Figure S1, Supplemental Content, which demonstrates the bias assessment of randomized controlled studies). The potential publication bias was tested by conducting of funnel plot, where the dotted vertical line represents the combined effect size in this outcome.

### Data Synthesis and Statistical Analysis

The risk ratios of any stroke/death, TIA, and restenosis were pooled across studies and analyzed using the Mantel–Haenszel statistical method to compare CAS with CEA. The amount of heterogeneity was estimated using *I*^2^ statistics, which uses values from 0 to 100% (0–24%, low heterogeneity; 25–49%, moderate heterogeneity; 50–74%, high level of heterogeneity; 75–100%, extreme heterogeneity). Random-effect meta-analysis models were chosen when heterogeneity > 50%, and fixed-effect models when heterogeneity < 50%.

All analyses were performed using the Cochrane Collaboration Review Manager (Version 5.20, Cochrane Collaboration, Copenhagen, Denmark). The probability values were two-tailed and the null hypothesis was rejected for values of *P* < 0.05.

## RESULTS

### Study Selection and Characteristics

**FIGURE 1:**
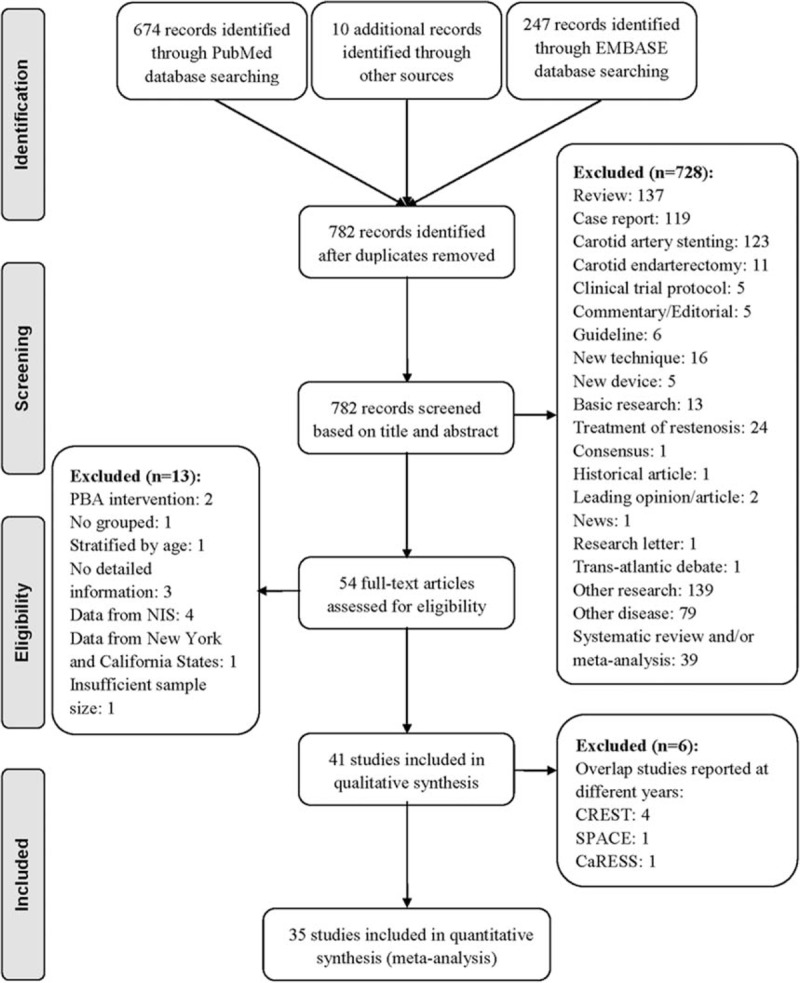
Flow diagram according to the Preferred Reporting Items for Systematic Reviews and Meta-Analyses guidelines. CaRESS = carotid revascularization using endarterectomy or stenting systems, CREST = carotid revascularization endarterectomy versus stenting trial, NIS = nationwide inpatient sample, PBA = percutaneous balloon angioplasty, SPACE = stent-supported percutaneous angioplasty of the carotid artery versus endarterectomy.

The literature search identified 782 potentially relevant studies, as shown in the flow diagram (Figure 1). Of these, 54 full-text articles were assessed for eligibility, and 41 studies met the inclusion criteria. Six studies that were the same but were reported in different years were excluded as follows: four “Carotid Revascularization Endarterectomy versus Stenting Trial (CREST),”^3,12–14^ one “Stent-Supported Percutaneous Angioplasty of the Carotid Artery versus Endarterectomy (SPACE)”,^15^ and one “Carotid Revascularization Using Endarterectomy or Stenting Systems (CaRESS).”^16^ Finally, 35 studies comprising 27,525 patients treated between January 1997 and March 2012 were included in the meta-analysis. There were 12 randomized controlled trials,^17–28^ three prospective controlled studies,^29–31^ and 20 retrospective comparative studies.^32–51^

The patients’ characteristics and comorbidities were summarized (see Table S2, Supplemental Content, which demonstrates the patients’ characteristics and comorbidities). The average age was 70 years, and 68.0% of patients were men. The comorbidities were hypertension (77.7%), coronary artery disease (40.4%), dyslipidemia (55.6%), diabetes mellitus (29.2%), and smoking (44.0%).

### Primary End Points

**FIGURE 2:**
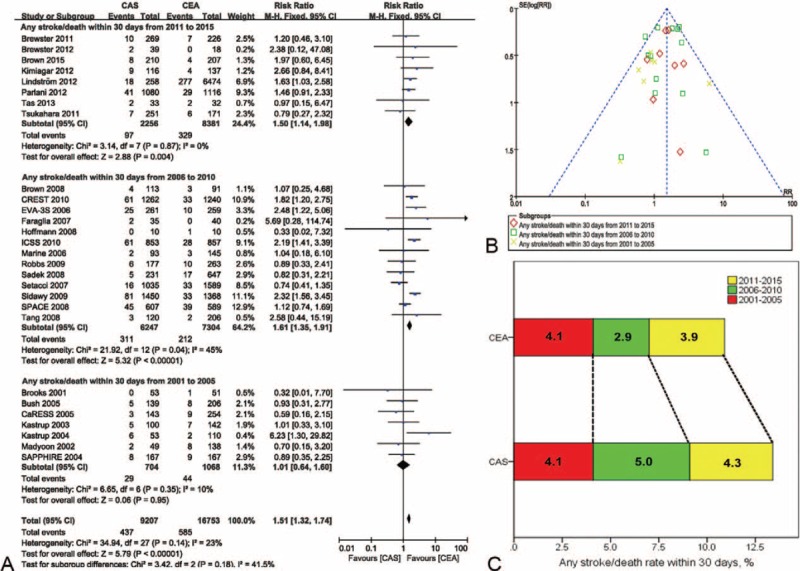
Meta-analysis of the stoke/death rate within 30 d at 5-year intervals. (A) The efficacy of CEA for freedom from stroke/death within 30 d was superior to that of CAS from 2006 to 2015. (B) The likelihood of publication bias was low. (C) The incidence rate of stroke/death within 30 d at 5-year intervals in CEA and CAS. CaRESS = carotid revascularization using endarterectomy or stenting systems, CAS = carotid artery stenting, CEA = carotid endarterectomy, CI = confidence interval (s), CREST = carotid revascularization endarterectomy versus stenting trial, EVA-3S = endarterectomy versus angioplasty in patients with symptomatic severe carotid stenosis, ICSS = international carotid stenting study, SAPPHIRE = stenting and angioplasty with protection in patients at high risk for endarterectomy, SPACE = stent-supported percutaneous angioplasty of the carotid artery versus endarterectomy.

**FIGURE 3:**
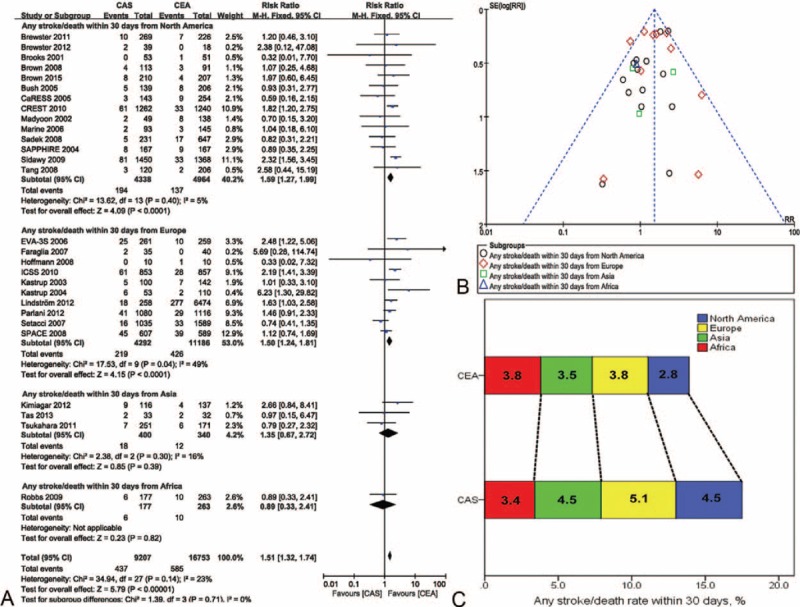
Meta-analysis of the stroke/death rate within 30 d worldwide. (A) The efficacy of CEA for freedom from stroke/death within 30 d was superior to that of CAS in North America and Europe. (B) The likelihood of publication bias was low. (C) The incidence rate of stroke/death within 30 d in different continents in CEA and CAS. CaRESS = carotid revascularization using endarterectomy or stenting systems, CAS = carotid artery stenting, CEA = carotid endarterectomy, CI = confidence interval(s), CREST = carotid revascularization endarterectomy versus stenting trial, EVA-3S = endarterectomy versus angioplasty in patients with symptomatic severe carotid stenosis, ICSS = international carotid stenting study, SAPPHIRE = stenting and angioplasty with protection in patients at high risk for endarterectomy, SPACE = stent-supported percutaneous angioplasty of the carotid artery versus endarterectomy.

The overall risk ratio of any stroke/death within 30 d of treatment was 1.51 (95% confidence interval [CI] 1.32–1.74, *P* < 0.001) with CAS versus CEA (Figures 2–4 A); there was low heterogeneity (I^2^ = 23%). The incidence of stroke/death within 30 d of treatment was 4.7% for CAS and 3.5% for CEA.

**FIGURE 4:**
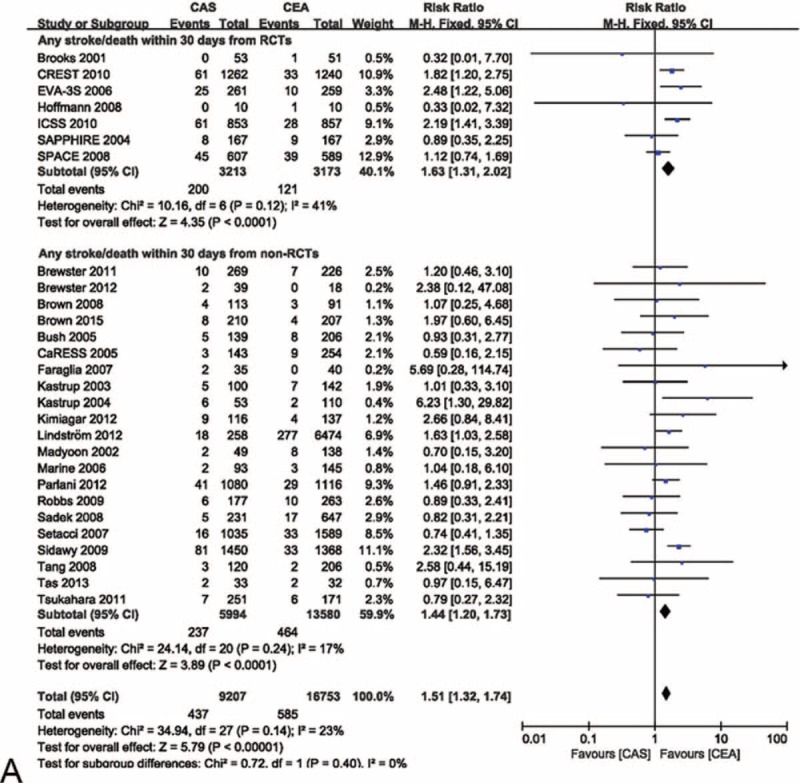
Meta-analysis of the stroke/death rate within 30 d from randomized and nonrandomized comparative studies. (A) The efficacy of CEA for freedom from stroke/death within 30 d was superior to that of CAS in randomized and nonrandomized comparative studies. (B) The likelihood of publication bias was low. CaRESS = carotid revascularization using endarterectomy or stenting systems, CAS = carotid artery stenting, CEA = carotid endarterectomy, CI = confidence interval(s), CREST = carotid revascularization endarterectomy versus stenting trial, EVA-3S = endarterectomy versus angioplasty in patients with symptomatic severe carotid stenosis, ICSS = international carotid stenting study, RCTs = randomized comparative studies, SAPPHIRE = stenting and angioplasty with protection in patients at high risk for endarterectomy, SPACE = stent-supported percutaneous angioplasty of the carotid artery versus endarterectomy.

**FIGURE 4 (Continued):**
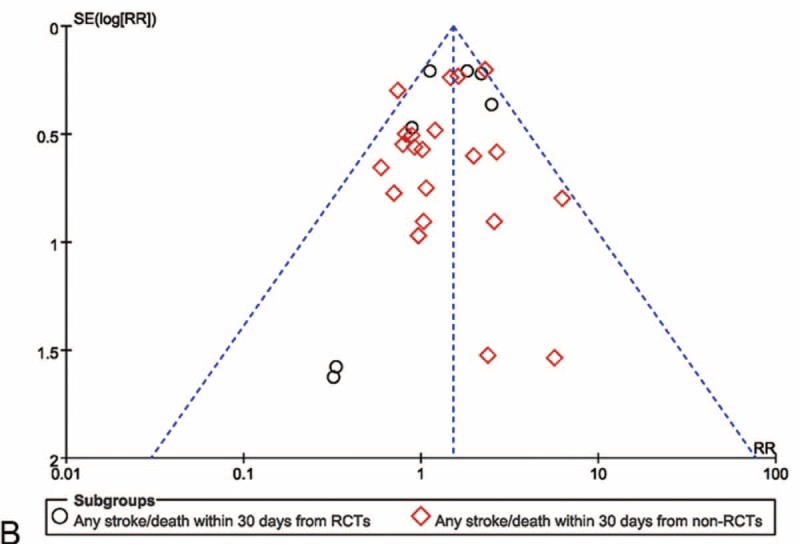
Meta-analysis of the stroke/death rate within 30 d from randomized and nonrandomized comparative studies. (A) The efficacy of CEA for freedom from stroke/death within 30 d was superior to that of CAS in randomized and nonrandomized comparative studies. (B) The likelihood of publication bias was low. CaRESS = carotid revascularization using endarterectomy or stenting systems, CAS = carotid artery stenting, CEA = carotid endarterectomy, CI = confidence interval(s), CREST = carotid revascularization endarterectomy versus stenting trial, EVA-3S = endarterectomy versus angioplasty in patients with symptomatic severe carotid stenosis, ICSS = international carotid stenting study, RCTs = randomized comparative studies, SAPPHIRE = stenting and angioplasty with protection in patients at high risk for endarterectomy, SPACE = stent-supported percutaneous angioplasty of the carotid artery versus endarterectomy.

The risk ratios of any stroke/death within 30 d were 1.50 (95% CI 1.14–1.98, *P* = 0.004) from 2011 to 2015, 1.61 (95% CI 1.35–1.91, *P* < 0.001) from 2006 to 2010, and 1.01 (95% CI 0.64–1.60, *P* = 0.95) from 2001 to 2005 when CAS was compared with CEA. There were low heterogeneity (I^2^ = 0%, 45%, and 10%, respectively) (Figure 2A). A funnel plot showed no significant evidence of asymmetry (Figure 2B). The incidence rates for CAS and CEA were 4.3% and 3.9% from 2011 to 2015, 5.0% and 2.9% from 2006 to 2010, and 4.1% for both from 2001 to 2005, respectively (Figure 2C).

The risk ratios of any stroke/death within 30 d of CAS versus CEA were 1.59 (95% CI 1.27–1.99, *P* < 0.001) in North America, 1.50 (95% CI 1.24–1.81, *P* < 0.001) in Europe, and 1.35 (95% CI 0.67–2.72, *P* = 0.39) in Asia. Heterogeneity was 5%, 49%, and 16%, respectively, for North America, Europe, and Asia. There was only one study from Africa,^42^ and the risk ratio was 0.89 (95% CI 0.33–2.41, *P* = 0.82) (Figure 3A). No significant evidence of asymmetry was observed in the funnel plot (Figure 3B). The incidence rates for CAS versus CEA were 4.5% versus 2.8% in North America, 5.1% versus 3.8% in Europe, 4.5% versus 3.5% in Asia, 3.4% versus 3.8% in Africa (Figure 3C).

The risk ratios of any stroke/death within 30 d of the randomized and nonrandomized comparative studies were 1.63 (95% CI 1.31–2.02, *P* < 0.001) and 1.44 (95% CI 1.20–1.73, *P* < 0.001), respectively. Heterogeneity was 41% and 17%, respectively, for the randomized and nonrandomized comparative studies (Figure 4 A). There was no significant evidence of asymmetry (Figure 4 B).

### Secondary End Points

**FIGURE 5:**
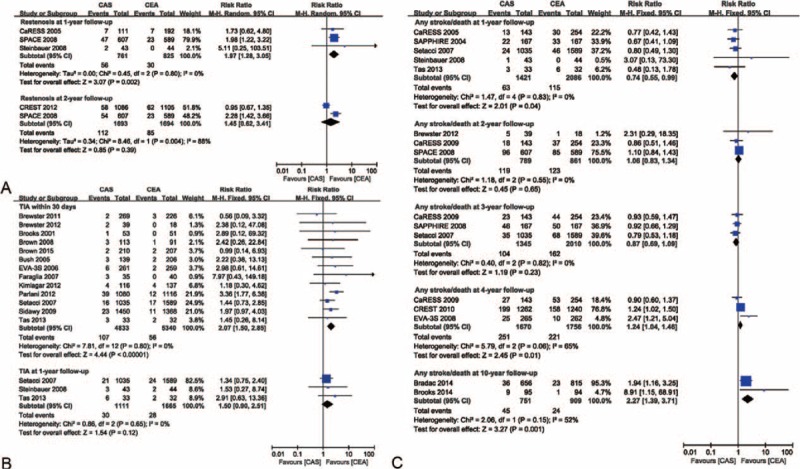
Meta-analyses of restenosis, transient ischemic attack, and stroke/death rates at different follow-up points. (A) The efficacy of CEA for freedom from restenosis was superior to that of CAS at 1-year follow-up. (B) The efficacy of CEA for freedom from transient ischemic attack was superior to that of CAS at 30 d. (C) The efficacy of CEA for freedom from stroke/death was superior to that of CAS at 4- and 10-year follow-up, but inferior to CAS at 1-year follow-up. CaRESS = carotid revascularization using endarterectomy or stenting systems, CAS = carotid artery stenting, CEA = carotid endarterectomy, CI = confidence interval(s), CREST = carotid revascularization endarterectomy versus stenting trial, EVA-3S = endarterectomy versus angioplasty in patients with symptomatic severe carotid stenosis, SAPPHIRE = stenting and angioplasty with protection in patients at high risk for endarterectomy, SPACE = stent-supported percutaneous angioplasty of the carotid artery versus endarterectomy, TIA = transient ischemic attack.

The risk ratios of restenosis at follow-up were 1.97 (95% CI 1.28–3.05, *P* = 0.002) after 1 year and 1.45 (95% CI 0.62–3.41, *P* = 0.39) after 2 year. Heterogeneity was 0% and 88%, respectively, after 1 and 2 year. The forest plot showed that the efficacy of CEA was superior to that of CAS at the 1-year follow-up point. The incidence rates for CAS and CEA were 7.4% and 3.6% at 1 year, and 6.6% and 5.0% at 2-year follow-up, respectively (Figure 5A). The funnel plot showed no significant evidence of asymmetry (see Figure S2A, Supplemental Content, which demonstrates the funnel plot for publication bias assessment of restenosis rate).

The risk ratios of TIA at 30 d and 1 year were 2.07 (95% CI 1.50–2.85, *P* < 0.001) and 1.50 (95% CI 0.90–2.51, *P* = 0.12), respectively, when CAS was compared with CEA. Heterogeneity was 0 for both. The forest plot showed that the efficacy of CEA was superior to that of CAS at 30 d. The incidence rates for CAS versus CEA were 2.2% versus 1.0% within 30 d and 2.7% versus 1.7% at 1-year follow-up, respectively (Figure 5B). No significant evidence of asymmetry was observed in the funnel plot (see Figure S2B, Supplemental Content, which demonstrates the funnel plot for publication bias assessment of TIA rate).

The risk ratios of any stroke/death were 0.74 (95% CI 0.55–0.99, *P* = 0.04) for 1-year, 1.06 (95% CI 0.83–1.34, *P* = 0.65) for 2-year, 0.87 (95% CI 0.69–1.09, *P* = 0.23) for 3-year, 1.24 (95% CI 1.04–1.46, *P* = 0.01) for 4-year, and 2.27 (95% CI 1.39–3.71, *P* = 0.001) for 10-year follow-up. Heterogeneity varied over the years (I^2^ = 0, 0, 0, 65%, and 52%, respectively). The forest plot shows that the efficacy of CEA was inferior to that of CAS at 1-year follow-up but was superior to that of CAS at 4- and 10-year follow-up examinations. The incidence rates for CAS and CEA were 4.4% and 5.5% at 1-year, 15.1% and 14.3% at 2-year, 7.7% and 8.1% at 3-year, 15.0% and 12.6% at 4-year, 6.0% and 2.6% at 10-year follow-ups, respectively (Figure 5C). There was no significant evidence of asymmetry (see Figure S2C, Supplemental Content, which demonstrates the funnel plot for publication bias assessment of stroke/death rate).

## DISCUSSION

Although many meta-analyses comparing CAS with CEA for carotid stenosis have been performed, there are disparities among the results.^4–9^ Subgroup analyses divided by age,^9^ anesthesia type,^52^ time,^53^ or symptom^54^ were helpful to determine the best therapeutic strategy under different circumstances. To our knowledge, the present study was the first meta-analysis to take the timeframes and worldwide differences into account.

CEA was found to be superior to CAS in freedom from stroke/death within 30 d of treatment, a finding that was different from that of previous studies.^55,56^ The superiority was significant from 2006 to 2015 but not from 2001 to 2005 based on the 5-year interval analyses. CEA was introduced as an effective treatment option to prevent stroke in the early 1950 s, whereas CAS provided a less-invasive option until 1994.^3^ As with any other endovascular or surgical procedure, common sense suggests that operator skills and experience have a major impact on CAS outcomes. The procedural stroke and death rates decreased over time and differed according to the level of operator experience.^57,58^ It is presumed that the criteria for the initial CAS procedures were simple and uncomplicated, which might explain the lower stroke/death rates from 2001 to 2005. Meanwhile, many surgeons experienced the first 100 patients for CEA and improved the treatment effects.^59^ An emboli-protection device effectively reduces the stroke/death rate^60,61^ and was recommended with the CAS procedure.^62,63^ The occurrence of stroke/death within 30 d decreased from 5.0% (2006–2010) to 4.3% (2011–2015) for CAS. This improvement in the clinical outcomes of the CAS procedure was associated with the use of an emboli-protection device.^64^ Because these devices were used more often, it is presumed that they are related to the positive postsurgery improvements.

The occurrence of stroke/death within 30 d for CAS patients was significantly higher than that for CEA patients in North America and Europe. As discussed previously, the first successful CEA was done by DeBakey in 1953.^3,65^ The innovative and effective technique spread rapidly and was adopted throughout the United States, Europe, Asia, Africa, and other parts of the world. The risk of stroke/death was lower in the headstream of the technique. In fact, > 96.6% patients who underwent CEA in the present meta-analysis were from North America and Europe. Any adverse effects from the procedure were associated with operator skills as well as the operation method itself. Having more patients in need of this treatment helped to raise the proficiency level of the surgeons and decreased the adverse effects from the procedure.

The incidence rate of TIA within 30 d was pronouncedly higher in CAS than in CEA. It is presumed that the complications are relevant to the procedure, in which the wire must pass through the atherosclerotic lesion with severe stenosis or total occlusion. On the other hand, the complications might be associated with the stent design. Carotid stents are now made of nitinol and available in closed-cell and open-cell designs. Although the closed-cell design might confer better plaque coverage than the open-cell design from a conceptual perspective, it still incises the plaque and causes many small emboli. The incidence rate of TIA is in accordance with the stroke/death rate within 30 d. Recent studies have demonstrated that overall survival is significantly lower in patients with postoperative TIA, which is an independent predictor of decreased survival at the 5-year follow-up.^66^

Restenosis is one of the main drawbacks of endovascular treatment of carotid stenosis, which would no doubt influence the therapeutic effect, especially for long lesions. Following stent deployment, inflation of the stent using a balloon catheter is mandatory. Nevertheless, neointimal hyperplasia and hemodynamic turbulence increase the possibility of in-stent restenosis.^67,68^ The restenosis rate in CAS is apparently higher than that in CEA at 1-year follow-up; however, the stroke/death rate in CAS is lower than that in CEA during the same time period. These conflicting results might be because of collateral compensatory circulation after CAS intervention. The advantage disappeared at 4- and 10-year follow-up examinations.

In the present study, 31 studies (95.7% patients) were from North America and Europe, whereas only three studies (2.7% patients) were from Asia and one (1.6% patients) from Africa. Scientific research has been guided by North America and Europe for many years. It should be noted that Asia, Africa, and other continents should strengthen their scientific research because their populations account for > 80% of the world population.

### Study Limitations

The systematic review and meta-analysis has some limitations. First, the subgroups were stratified by the publication year, not the year of patients enrolled in. On the other hand, the subgroups were divided by the location in which the study was mainly performed, which was not rigorous to the multicenter, intercontinental studies. Second, the meta-analysis included prospective randomized controlled trials, prospective controlled studies, as well as retrospective comparative studies, which might lower the evidence level of the results. However, heterogeneity was low among the studies. Third, many confounding factors such as lesion length, stent types (closed-cell or open-cell, with or without emboli protection devices), methods of endarterectomy (conventional or eversion, with or without patch), antiplatelet therapy, and clinical manifestation of patients (symptomatic or asymptomatic) were not considered in the present study.

## CONCLUSIONS

The current published body of literature suggests that the efficacy of CEA is superior to that of CAS for freedom from the stroke/death rate within 30 d of the procedures, especially that from 2006 to 2015 and in North America and Europe. The superiority of CEA over CAS was also observed for the restenosis rate at 1-year, TIA rate within 30 d, and stroke/death rate at 4- and 10-year follow-up examinations. On the contrary, the efficacy of CEA is inferior to that of CAS for the stroke/death rate at 1-year follow-up.
