# Systematic Review and Meta-Analysis of Carotid Artery Stenting Versus Endarterectomy for Carotid Stenosis: A Chronological and Worldwide Study: Erratum

**DOI:** 10.1097/MD.0000000000009591

**Published:** 2018-01-05

## Abstract

Supplemental Digital Content is available in the text

In the article, “Systematic Review and Meta-Analysis of Carotid Artery Stenting Versus Endarterectomy for Carotid Stenosis: A Chronological and Worldwide Study”^[1]^, the supplemental digital content links were left out. These sentences should have appeared as:

There were no restrictions on the year or the type of publication. The search strategy was amended for each database (see Table S1, Supplemental Content, which demonstrates the search strategies for PubMed and Embase databases).

The Cochrane Collaboration risk of bias tool was used to assess the quality of included randomized controlled trials (see Figure S1, Supplemental Content, which demonstrates the bias assessment of randomized controlled studies).

The patients’ characteristics and comorbidities were summarized (see Table S2, Supplemental Content, which demonstrates the patients’ characteristics and comorbidities).

The funnel plot showed no significant evidence of asymmetry (see Figure S2A, Supplemental Content, which demonstrates the funnel plot for publication bias assessment of restenosis rate).

No significant evidence of asymmetry was observed in the funnel plot (see Figure S2B, Supplemental Content, which demonstrates the funnel plot for publication bias assessment of TIA rate).

There was no significant evidence of asymmetry (see Figure S2C, Supplemental Content, which demonstrates the funnel plot for publication bias assessment of stroke/death rate).

## Supplementary Material

Supplemental Digital Content
