# Supplementary material for: Systematic Review and Meta-Analysis of Carotid Artery Stenting Versus Endarterectomy for Carotid Stenosis: A Chronological and Worldwide Study: Erratum
Source: Medicine (Baltimore). 2018 Jan 5;97(1):e9591. doi: 10.1097/MD.0000000000009591 (PMC5943111; doi:10.1097/MD.0000000000009591)
Supplement: Supplemental Digital Content [file medi-97-e9591-s001.pdf]

Zhang L, et al. **Systematic review and meta-analysis of carotid artery stenting versus endarterectomy for carotid stenosis: a chronological and worldwide study**

Figure S1. Bias assessment of randomized controlled studies

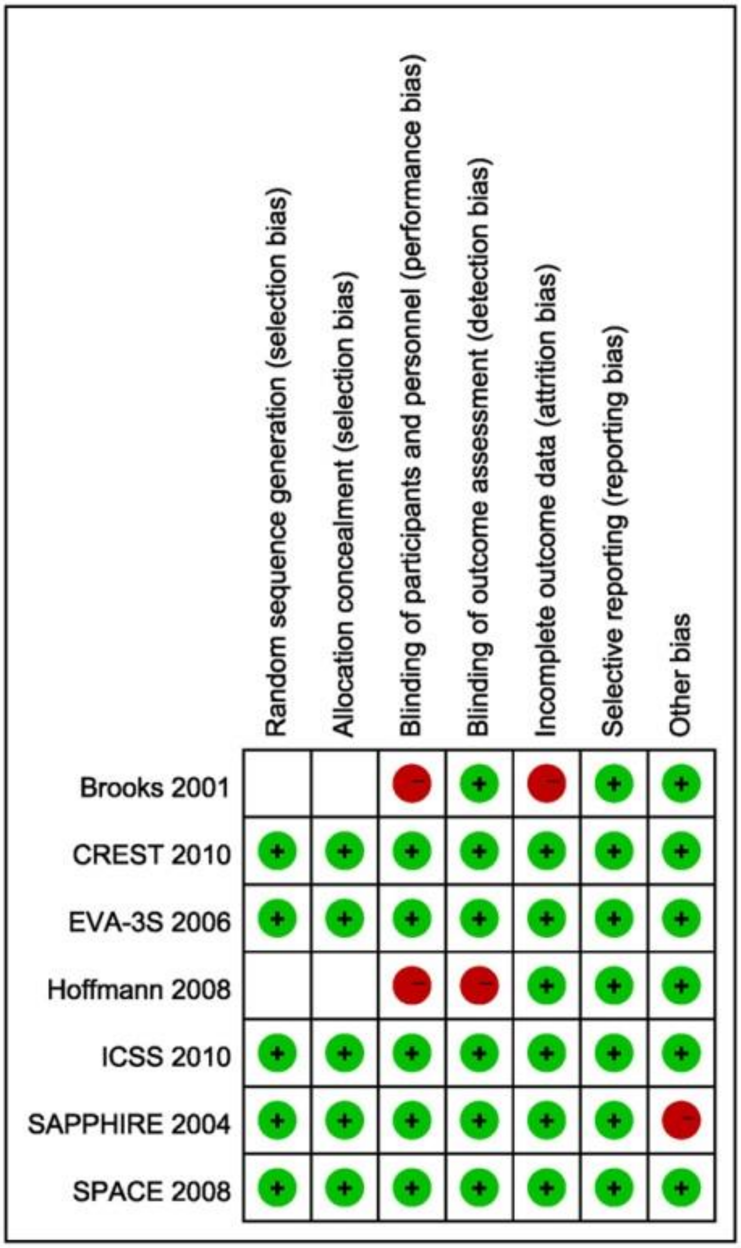

Zhang L, et al. **Systematic review and meta-analysis of carotid artery stenting versus endarterectomy for carotid stenosis: a chronological and worldwide study**

Figure S2. Funnel plots for publication bias assessments of restenosis, transient ischemic attack and stroke/death rates

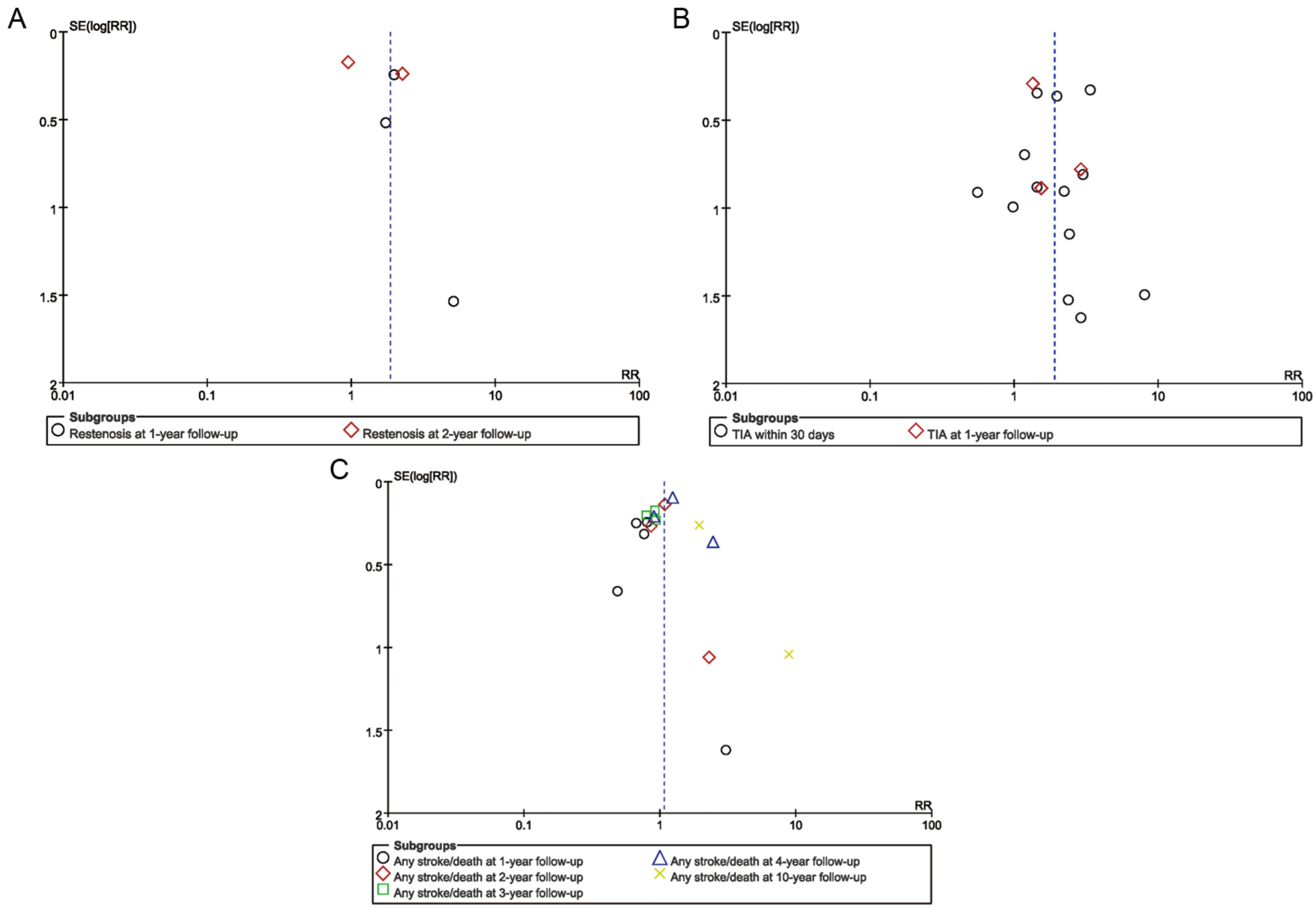

Table S1. Search strategies in PubMed and Embase databases (February 4, 2015)

PubMed

Search	Query	Items found	Time
#6	Search #1 AND #2 AND #3 AND #4 AND #5 Filters: Humans; English	674	20:16:21
#5	Search (carotid arteries[MeSH Terms]) OR carotid stenosis[MeSH Terms] Filters: Humans; English	33910	20:12:22
#4	Search ((CEA[Title/Abstract]) OR carotid endarterectomy[Title/Abstract]) OR surgical[Title/Abstract]Filters: Humans; English	443182	20:11:25
#3	Search ((CAS[Title/Abstract]) OR carotid artery stenting[Title/Abstract]) OR endovascular[Title/Abstract] Filters: Humans; English	26758	20:10:54
	Search (stenosis[Title/Abstract]) OR occlusion[Title/Abstract] Filters: Humans; English	120290	20:10:17
#1	Search carotid arter*[Title/Abstract] Filters: Humans; English	29130	20:09:25

Embase		
**Search**	**Query**	**Items found**
#6	#1 AND #2 AND #3 AND #4 AND #5	247
#5	'carotid artery'/exp	67310
#4	'cas':ab,ti OR 'carotid artery stenting':ab,ti OR endovascular:ab,ti AND ([article]/lim OR [article in press]/lim) AND [humans]/lim AND [english]/lim AND [embase]/lim	22984
#3	'cea':ab,ti OR 'carotid endarterectomy':ab,ti OR surgical:ab,ti AND ([article]/lim OR [article in press]/lim) AND [humans]/lim AND [english]/lim AND [embase]/lim	314442
#2	stenosis:ab,ti OR occlusion:ab,ti AND ([article]/lim OR [article in press]/lim) AND [humans]/lim AND [english]/lim AND [embase]/lim	90104
#1	'carotid'/exp OR carotid AND arter*:ab,ti AND ([article]/lim OR [article in press]/lim) AND [humans]/lim AND [english]/lim AND [embase]/lim	32111

Zhang L, et al. **Systematic review and meta-analysis of carotid artery stenting versus endarterectomy for carotid stenosis: a chronological and worldwide study**

Table S2 Baseline characteristics of all included studies subjects

Study	Year	Location	Time	N	Age	Male	HTN	CAD	DLM	DM	Smoking
Ref 32	2015	USA	2007-2012	210/207	68/70	134/127	188/192	NR/NR	153/160	74/69	67/67
Ref 17, 28	2014, 2001	USA	NR	53/51	66/70	NR	45/48	39/31	34/24	19/12	38/40
Ref 33	2014	CR	2003-2012	656/815	70/66	485/575	NR	NR	NR	NR	NR
Ref 34	2013	Turkey	2011-2012	33/32	72/70	25/26	25/21	23/16	NR	11/20	20/25
Ref 35	2012	Italy	2001-2009	1080/1116	72/71	767/791	903/887	397/296	671/580	NR	NR
Ref 36	2012	Sweden	2004-2011	258/6,474	69/NR	192/NR	173/NR	NR	NR	61/NR	81/NR
Ref 37	2012	Israel	1999-2007	116/137	69/68	91/102	91/83	81/51	61/60	58/55	30/54
Ref 38	2012	USA	2007-2011	39/18	67/70	28/12	32/18	6/2	27/13	16/5	14/5
Ref 18, 20	2012, 2010	USA	2000-2008	1262/1240	69/69	806/823	1083/1068	NR	1046/1064	386/377	333/324
Ref 39	2011	Japan	2001-2009	251/171	71/70	80/82	40/70	10/19	21/20	18/24	NR
Ref 40	2011	USA	2007-2010	269/226	70/70	189/185	245/212	NR	225/185	108/79	NR
Ref 19	2010	Europe	2001-2008	853/857	70/70	601/606	587/595	NR	522/562	184/187	205/198
Ref 41	2009	USA	-2007.12	1450/1368	71/71	863/817	1183/1078	890/625	NR	478/357	860/761
Ref 42	2009	SA	2005-2008	177/263	NR	NR	152/153	50/83	22/28	71/90	61/119
Ref 29, 30	2009, 2005	USA	NR	143/254	71/71	86/161	116/206	95/154	91/177	42/61	NR
Ref 43	2008	USA	2001-2006	120/206	NR	NR	106/169	8/10	84/138	38/60	19/53
Ref 21	2008	Germany	1999-2002	43/44	68/68	NR	34/34	18/20	22/23	19/15	19/18

Ref 44	2008	USA	1997-2002	231/647	NR	NR	202/495	NR	155/344	NR	NR
Ref 22	2008	Switzerland	1998-2002	10/10	69/71	8/9	7/8	2/4	7/6	3/3	5/6
Ref 24	2008	Germany	2001-2006	607/589	68/69	436/422	455/449	127/142	NR	157/169	428/412
Ref 45	2008	USA	1997-2006	113/91	70/67	111/89	106/82	21/29	NR	32/30	95/81
Ref 25, 26	2008, 2006	France	2000-2005	265/262	69/70	193/204	192/188	NR	154/146	59/67	65/61
Ref 23, 27	2008, 2004	USA	2000-2002	167/167	73/73	112/112	143/142	143/126	131/128	42/46	28/27
Ref 46	2007	Italy	2000-2006	1035/1589	74/65	NR	736/1164	458/706	285/410	321/524	605/838
Ref 47	2007	Italy	2005-2007	35/40	73/67	22/32	29/33	9/11	16/8	11/6	25/34
Ref 48	2006	USA	2003-2005	93/145	NR	NR	85/112	69/84	70/97	35/44	54/79
Ref 49	2005	USA	2001-2004	139/206	68/66	125/196	119/150	89/66	48/73	41/71	104/159
Ref 50	2004	Germany	1997-2004	53/110	78/78	36/70	45/87	13/23	26/34	9/33	2/8
Ref 51	2003	Germany	1999-2001	100/142	70/70	75/105	86/118	18/30	48/63	26/46	30/42
Ref 31	2002	USA	NR	49/138	74/72	NR/80	NR	NR	NR	NR	NR
Sum, n (%)	NA	NA	NA	27 525	70 (Mean)	11091 (68.0)	15070 (77.7)	5094 (40.4)	8262 (55.6)	4769 (29.2)	6599 (44.0)

The data were presented as CAS/CEA.

CAD = coronary artery disease, CAS = carotid artery stenting, CEA = carotid endarterectomy, CR = Czech Republic, DM = diabetes mellitus, DLM = dyslipidemia, HTN = hypertension, NA = not applicable, NR = not reported, SA = South Africa, USA = the United States of America.
